# Editorial Notes

**Published:** 1883-04

**Authors:** 


### EDITORIAL NOTES.

THE Association of American Medical Editors will hold its next meeting in the city of Cleveland, simultaneously with that of the American Medical Association, June 5th and 6th, 1883. The address will be delivered by Dr. N. S. Davis, of Chicago, on "The Present Status and Tendencies of the Medical Profession and Medical Journalism." Dr. J. V. Shoemaker, the able Secretary, has issued the call, and we hope to be able to attend.

THE *Journal of Nervous and Mental Diseases*, published by G. P. Putnam's Sons, and edited by W. J. Morton, M. D., is among the most valuable of our exchanges, as it is the most complete and able of the medical periodicals in the special department to which it is devoted. To those of our readers who desire the best thoughts of our ablest neurologists, we recommend this journal. Its appearance in type and paper corresponds with all the publications issued by the enterprising publishers.

WE have received, through the kindness of Dr. Fell, of the Committee on Publication, a copy of the Proceedings of the American Society of Microscopists, at its fifth annual meeting, which was held

August 15, 1882, at Elmira, N. Y. It is printed on fine tinted paper and contains 292 pages of valuable matter, presented in a form which is highly creditable to the society. The publishers are Messrs. Bigelow Brothers, of this city, and the typographical execution of the work reflects much credit upon their taste. There is a great variety of subjects included in the work, many of them being of professional interest. We notice a very interesting paper by Dr. Holbrook on the Termination of the Nerves in the Liver; a paper by Dr. Ephraim Cutler on the Vegetable Nature of Croup; while many other of the papers show great labor in the special field of microscopy to which the meeting was principally devoted. The proceedings are a valuable addition to the literature of this interesting department of scientific research, and we are gratified to notice this evidence of proficiency of which the present volume is a highly creditable expression.

We have been favored with a copy of the *Bulletin* of the Buffalo Naturalists' Field Club, published under the editorial management of Professors Kellicott and Fish and Mrs. Dr. Moody. The object of this periodical is to publish brief résumés of general papers read at the winter meetings of the club, with short, original papers or memoirs on all branches of natural science, and particularly notes on the occurrence and habits of local plants and animals. The *Bulletin* contains a list of members of the club, with a brief history of the organization, the Constitution and By-laws, and several very interesting papers. We notice with pleasure a paper on the "Nesting Habits of Birds," by E. E. Fish, of this city, who finds delightful recreation from school-work in watching the habits of birds. Prof. Kellicott gives a very readable paper, entitled "Notes on Protozoa." The dissemination of plants is treated pleasantly by Miss Edna M. Procter. Mr. John F. Cowell treats of "Adventives at East Buffalo, and Miss Annie W. McMillan of the "Ornamental Features of our Trees and Shrubs," while Prof. Linden discusses the "Field Club Work of Western New York." Botanical, entomological and geological notes complete the number. The paper and type are excellent, and the *Bulletin* commands respect not only from its appearance but by the value and interest of its contents.

